# Improving Bioprocess Conditions for the Production of Prodigiosin Using a Marine *Serratia rubidaea* Strain

**DOI:** 10.3390/md22040142

**Published:** 2024-03-23

**Authors:** Ricardo F. S. Pereira, Carla C. C. R. de Carvalho

**Affiliations:** 1 Department of Bioengineering, iBB—Institute for Bioengineering and Biosciences, Instituto Superior Técnico, Universidade de Lisboa, Av. Rovisco Pais, 1049-001 Lisboa, Portugal; ricardofspereira@tecnico.ulisboa.pt; 2 Associate Laboratory i4HB—Institute for Health and Bioeconomy, Instituto Superior Técnico, Universidade de Lisboa, Av. Rovisco Pais, 1049-001 Lisboa, Portugal

**Keywords:** *Serratia rubidaea*, *Serratia marcescens*, medium engineering, *k_L_a*, bioreactors

## Abstract

The enormous potential attributed to prodigiosin regarding its applicability as a natural pigment and pharmaceutical agent justifies the development of sound bioprocesses for its production. Using a *Serratia rubidaea* strain isolated from a shallow-water hydrothermal vent, optimization of the growth medium composition was carried out. After medium development, the bacterium temperature, light and oxygen needs were studied, as was growth inhibition by product concentration. The implemented changes led to a 13-fold increase in prodigiosin production in a shake flask, reaching 19.7 mg/L. The conditions allowing the highest bacterial cell growth and prodigiosin production were also tested with another marine strain: *S. marcescens* isolated from a tide rock pool was able to produce 15.8 mg/L of prodigiosin. The bioprocess with *S. rubidaea* was scaled up from 0.1 L shake flasks to 2 L bioreactors using the maintenance of the oxygen mass transfer coefficient (*k_L_a*) as the scale-up criterion. The implemented parameters in the bioreactor led to an 8-fold increase in product per biomass yield and to a final concentration of 293.1 mg/L of prodigiosin in 24 h.

## 1. Introduction

The growing need for compounds capable of addressing multiple health problems like antibiotics for multi-resistant strains and antitumor and anticancer agents has stimulated the quest for interesting bioactive natural products from marine bacteria. Prodigiosin, a lead compound of the 4-methoxypyrrolyldipyrrin family of natural products, presents immunosuppressive and anticancer activities, besides antifungal, antibacterial, antiprotozoal and antimalarial activities [1,2,3,4]. For its anticancer activity, a new anticancer therapy with prodigiosin/PU-H71 could be used as a combined therapy for triple-negative breast cancer [5]. Recently, prodigiosin was also proven effective in controlling harmful algal blooms by acting as an algicide against *Heterosigma akashiwo* [6] and in regulating *Microcystis* blooms and inhibiting the production of their toxins, microcystins [7]. Additionally, the application of prodigiosin to fabrics provided antimicrobial properties to the textiles which could be used in hospitals to decrease hospital-acquired infections [8], while contributing to the stability, durability and biodegradation of the coated fabric [9].

Since the chemical synthesis of prodigiosin has many steps, is costly and has a low rate when compared to the biosynthetic routes [10], microbial production may be regarded as the best route for future industrialization. To guarantee prodigiosin availability for multiple biotechnological applications, the development of bioprocesses that can lead to the production of high titers of this red compound is paramount. To reach this objective, medium composition and production conditions must be carefully selected and optimized [11]. Using medium engineering, the type, source, concentration and proportion of essential nutrients required to improve biomass and/or product concentrations are optimized while cell viability is maintained and production time and costs and downstream processing complexity are reduced [12,13]. After the medium composition is optimized, the bioprocess performance often increases up to 60% [14].

Studies related to medium composition usually use an empirical approach that involves the testing of different ingredients and compositions resulting in numerous experiments, making the process often laborious, time-consuming and expensive [15,16]. However, in industry, it is still a common practice during bioprocess development, every time a new strain or mutant is used [14]. In fact, designing a defined medium is considered a good practice in industry, regardless of the increase in cost, since the medium composition promptly influences the performance of microbial processes for every new supplement added [17]. This rise in cost is often justified by the higher concentration in biomass and/or product yield, biomanufacturing reproducibility and simplification of the regulatory paperwork [18,19].

Regarding prodigiosin producers, various bacterial genera from both marine and terrestrial environments have been found, with *Serratia marcescens* being the best-studied species [20,21,22]. However, it is known that *S. marcescens* cells may cause both opportunistic and nosocomial infections, as well as other infections such as pneumonia, endocarditis and meningitis, in both children and adults [23,24,25]. Large-scale cultivation of *S. marcescens* is thus generally not regarded as safe. Additionally, since prodigiosin is a secondary metabolite of *S. marcescens*, it is only produced during the stationary phase and may take 36 to 96 h for the start of its production, which significantly increases production costs. Efforts have been made to transfer the pig genes responsible for prodigiosin biosynthesis in *S. marcescens* to Generally Recognized as Safe (GRAS) hosts such as *Pseudomonas putida* KT2440 which can be safely handled to upscale the bioprocess [26]. However, the antibacterial properties of prodigiosin and the low titers attained with the hosts reinforce that there is still a need for reliable and efficient strains for industrial processes able to produce the product on the gram scale [27].

In a previous paper, we reported the isolation of a *S. rubidaea* strain from a marine sample able to produce prodigiosin, and we have shown that Marine Broth (MB) allowed the highest production among several media tested [28]. The bacterium was isolated from a sample collected at a shallow-water hydrothermal vent and could produce prodigiosin up to a temperature of 62 °C. This could be an advantage for industrial production of prodigiosin, e.g., to prevent contamination from other bacteria. More importantly, this strain starts producing prodigiosin after 5 h of fermentation. This is probably an adaptative strategy to the extreme environment of the sampling site, since prodigiosin may provide photoprotection during low tide when direct exposure of the site to sunlight occurs.

The goal of the present work was to increase the production of prodigiosin by *S. rubidaea*, in shake flask, by supplementing MB medium with extra carbon (C), nitrogen (N) and metal ions, taking into consideration the natural habitat where this marine *S. rubidaea* was isolated from and the protein cluster for prodigiosin production. After the medium composition and the physical conditions were optimized, the process was scaled up to 2 L bioreactors using the maintenance of the oxygen mass transfer coefficient (*k_L_a*) as the scale-up criterion.

## 2. Results and Discussion

### 2.1. Enhancing Biomass and Product Production

#### 2.1.1. Effect of Carbon Sources

The first approach for improving bacterial growth and product production is to search for nutrients to be added to the culture medium that result in an increase in the concentration of both biomass and the metabolite of interest. The *S. rubidaea* used in this study was isolated from a shallow-water hydrothermal vent, and the preferred nutrients are difficult to be inferred.

**Figure 1:**
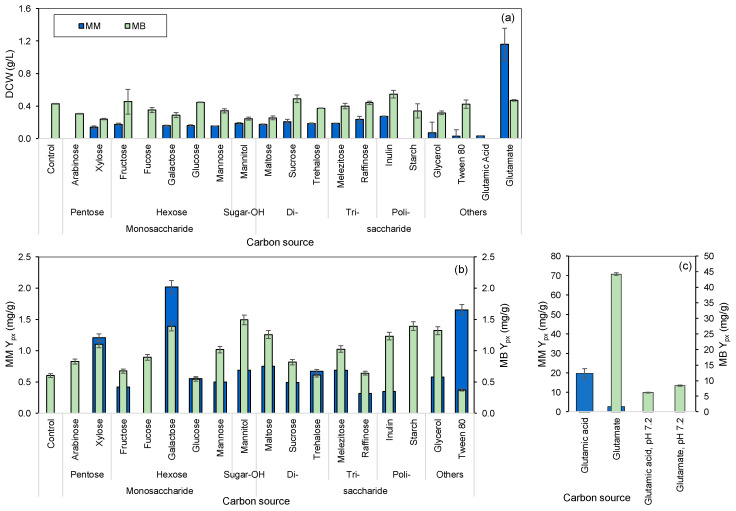
Dry cell weight (DCW; (**a**)) and product-to-biomass yield (Y_px_; (**b**,**c**)) when *S. rubidaea* cells were grown in MM and in MB supplemented with different sugars, saccharides and other carbon sources. Control represents the media without any carbon source supplementation.

To determine which carbon source would improve the bioprocess yields in the present study, isolated colonies of *S. rubidaea* from Marine Agar (MA) plates, were used to inoculate (i) mineral medium (MM) supplemented with 30 g/L of NaCl and (ii) marine broth (MB), both supplemented with different carbon sources. The composition of MB promoted, in general, both the growth of *S. rubidaea* cells and prodigiosin production when compared to MM which is a defined medium composed only of inorganic salts (Figure 1). However, the latter allowed the study of how the strain behaved in the presence of each individual compound assessed as a sole carbon source. Under carbon scarcity, bacteria can activate specific metabolic routes containing certain enzymes, such as α-glucosidase and inulinase, allowing them to use the available carbon compounds as carbon and energy sources [29]. The highest DCW values were attained in MM when the cells used glutamate, inulin and raffinose as carbon sources (Figure 1a). *S. rubidaea* cells did not grow in MM alone or supplemented with arabinose, fucose or starch. Nevertheless, in MB, the cells could grow in their presence and produce prodigiosin, indicating that they did not inhibit cell growth or production per se in the concentrations tested (Figure 1a).

The sugars xylose and galactose and the synthetic surfactant Tween 80 allowed the highest product per biomass yield (Y_px_) in MM (Figure 1b). In MB, *S. rubidaea* cells presented the highest Y_px_ values when the polysaccharides starch and inulin, the sugars galactose, mannitol and mannose, or glycerol were added to the medium. Mannitol is a sugar alcohol obtained by the reduction of mannose and may account for 20–30% of the dry weight of brown algae and could thus be an important source of carbon for heterotrophic bacteria in marine environments [30].

Media with amino acids as sole carbon sources have been used for several decades to promote bacterial growth [31,32], and glutamic acid, both in acidic and basic forms, has been shown to promote prodigiosin production [21]. Glutamic acid considerably increased the production of prodigiosin only in MM (Figure 1c). Sodium glutamate (NaG), its basic form, significantly promoted the production of prodigiosin in *S. rubidaea*, both in MM and MB, when compared to the other carbon sources resulting in high Y_px_. The Y_px_ was 16 times higher when glutamate was used in MB in comparison to MM, and 30 times higher when compared to the yield attained with mannitol, and glutamate was thus chosen as a supplement for MB. MM contained phosphate buffer, whereas commercial MB did not contain a buffering system. When glutamic acid was added to MB, the pH dropped to 3.6, which could explain why no growth or prodigiosin production was observed. When the pH was corrected to 7.2, Y_px_ with glutamic acid was 4-fold higher than with mannitol as a carbon source, but Y_px_ with glutamate decreased 5-fold in comparison to the cultivation in unbuffered MB. When glutamate was added to MB, the pH dropped to 6.3. As shown previously, pH has a high impact on prodigiosin production, and at pH 6.2, a ca. 3-fold increase in prodigiosin production was observed in comparison to pH 7 [28].

**Figure 2:**
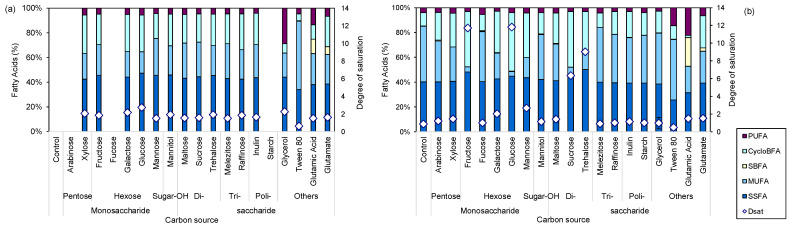
Fatty acid profile and corresponding degree of saturation of the membrane of *S. rubidaea* grown on different carbon sources in MM (**a**) and MB (**b**). Control represents the media without additional carbon source supplementation. Abbreviations: saturated straight fatty acid (SSFA), mono-unsaturated FA (MUFA), saturated branched FA (SBFA), cyclopropyl branched FA (CycloBFA), polyunsaturated FA (PUFA), and degree of saturation (Dsat).

Bacteria are able to modulate the fatty acid composition of the phospholipids of their cellular envelope as a response to, e.g., the carbon source used and the environmental conditions [33,34]. One of the parameters used to assess the changes is the degree of saturation, which is presented as the ratio between total saturated (saturated straight fatty acids—SSFAs) and unsaturated fatty acids (monounsaturated fatty acids—MUFAs). SSFAs and MUFAs are the most common FAs in the membrane of the present strain (Figure 2) and are the ones mainly responsible for its phase transition properties. A larger content of SSFAs results in higher rigidity of the membrane, whereas an increased content of MUFAs leads to increased membrane fluidity [35].

In MM, cells grown in the tested pentose and hexose sugars presented a cellular envelope containing, on average, 45% SSFAs, 23% MUFAs, 27% cyclopropyl branched fatty acids (CycloBFAs), and 5% polyunsaturated fatty acids (PUFAs; Figure 2a). The exception was observed with glucose, with the cells presenting a more rigid membrane as it contained 47% SSFAs and 17% MUFAs resulting in a ca. 30% higher degree of saturation than that observed for the cells grown in the other monosaccharides. In MB, these latter sugars induced the production of larger amounts of CycloBFAs than MUFAs: on average, the cells produced 43% SSFAs, 19% MUFAs, 34% CycloBFAs, and 4% PUFAs, with glucose- and fructose-grown cells presenting 45% SSFAs and only 4% MUFAs, resulting in a high degree of saturation of the cellular membrane (Figure 2b). The cells grown on MB supplemented with di-, tri-, and polysaccharides presented fatty acid profiles similar to those grown on MM with the same sugars: 42% SSFAs, 30% MUFAs, 25% CycloBFAs, and 3% PUFAs, with exceptions being observed with sucrose and trehalose (Figure 2b). With the latter disaccharides, the cells presented only 7 and 13% MUFAs, respectively, for sucrose and trehalose. Since sucrose is a disaccharide composed of glucose and fructose and trehalose contains two glucose molecules, the results corroborate those observed with these monosaccharides. Glycerol induced the production of PUFAs in the cellular membrane of *S. rubidaea* grown in MM, reaching 29% of the lipid content, but only 10% of that value was observed when the cells grew on MB (Figure 2). On the other hand, glutamate and especially glutamic acid induced the production of PUFAs and SBFAs in these cells. The content of SBFAs in cells grown with glutamic acid reached 23% in cells grown on supplemented MB and 11% in MM, while the content of PUFAs reached 22% in MB-grown cells and 13% in MM (Figure 2). Cells grown with the polysaccharide inulin presented similar fatty acid compositions in both MM and MB.

**Figure 3:**
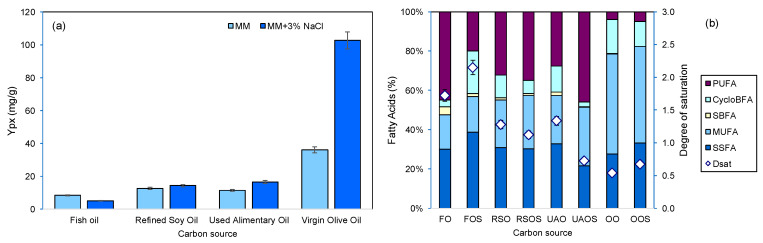
Product-to-biomass yield (**a**) and fatty acid composition of the cellular membrane of *S. rubidaea* cells and respective unsaturation index (**b**), when the cells were grown in mineral medium, with and without salt, with oils as carbon sources. Abbreviations: fish oil (FO), refined soy oil (RSO), used alimentary oil (UAO), olive oil (OO); “S” at the end of the name indicates the presence of 3% NaCl in the medium.

In the marine environment, the available carbon sources for microbial growth also include lipids and complex carbon sources, such as proteins, as a result of, e.g., fish decomposition, and their proportion may vary along the water column and sediments [36,37]. To determine if *S. rubidaea* cells could use each oil as a sole carbon source, cultivation was carried out in MM. When oils were used, in the presence and absence of salt, both the Y_px_ and the lipid profile of the cell membrane were changed in comparison to the results obtained with sugars (Figure 3). The lipids favored cell growth and product production in comparison with the sugar-based carbon sources in MB, with Y_px_ varying from 8.4 mg/g with fish oil to 36.2 mg/g in virgin olive oil (Figure 3a).

The presence of 3% NaCl, close to the average 3.5% of sea salt concentration, favored the overall production of both biomass and product, with the exception of fish oil which allowed a larger production of biomass resulting in a lower Y_px_ (Figure 3a). The fatty acid composition of olive oil resulted in higher yields of prodigiosin than those observed with the other tested oils, and a 2.8-fold increase in Y_px_ was attained when salt was added. The composition of refined oil and alimentary oil led to a prodigiosin per biomass yield that was higher than that with fish oil but much lower than that with virgin olive oil. Prodigiosin biosynthesis requires the synthesis of the precursor molecule 2-methyl-3-*n*-amyl-pyrrole catalyzed by PigD, PigB and PigE via pyruvate and 2-octenal, the latter being synthesized by fatty acid biosynthesis enzymes or by autooxidation of unsaturated fatty acids [38]. The existence of unsaturated fatty acids in the carbon source could thus favor prodigiosin production in *S. rubidaea.* Similar results have been observed with other *Serratia* strains with sunflower oil, olive oil, palm oil and peanut powder [39]. Prodigiosin, which has a higher affinity to hydrophobic organic compounds, formed small red dots in the salted MM medium, which resulted from the entrapment of the product inside the dispersed droplets of oil. Lin et al. reported the production of prodigiosin pellets by *S. marcescens* FZSF02 in a medium containing peanut power and olive oil [40].

The lipid profile of the cellular membrane of the cells grown in these oils was influenced by these carbon sources (Figure 3). Cells grown on fish, refined soy and used alimentary oils presented the largest amounts of PUFAs, whereas cells grown on olive oil contained mainly MUFAs. The incorporation of fatty acids from the carbon sources into the cells reduces the metabolic expenditure related to lipid synthesis which could be used for product production [41]. Additionally, the incorporation of PUFAs, uncommon in bacteria, will influence the organization of the lipid bilayer of membranes and may inhibit sodium ion channels [34,42]. Membrane pores similar to canonical voltage-gated sodium channels have been identified in *S. marcescens* [43]. In the present study, when the cells grew in fish oil, they significantly decreased the amount of PUFAs to 62.6% when salt was added to the medium (Figure 3). However, the converse was observed with other oils tested: an increase of 8.6, 4.3 and 47.0% was observed when the *S. rubidaea* cells grew in refined soy, used alimentary and olive oils, respectively. Apparently, the cells could adapt the lipid composition of their cell envelope as a response to the carbon source and NaCl in the medium.

As mentioned previously, the highest yield was attained with olive oil as the carbon source. However, there are several disadvantages from a process standpoint: (1) the high cost of olive oil; (2) in recent years, olive trees have been threatened with disease [44], making the acquisition of olive oil more expensive as production yields decrease; (3) the main market of olive oil is the competitive alimentary sector; and (4) the downstream process for purification of prodigiosin is more difficult and expensive. A comparison of the results shown in Figure 1c and Figure 3a indicates that the second highest product-to-biomass yield was attained with cells grown in MB with glutamate, reaching 44.2 mg/g. It is known that marine bacteria may accumulate amino acids, and glutamate in particular, for osmoregulation as a response to fluctuating sodium chloride concentrations in seawater [45]. From a bioprocess development perspective, MB supplemented with sodium glutamate was thus selected for the remaining studies carried out in this work.

#### 2.1.2. Effect of Nitrogen Sources

**Figure 4:**
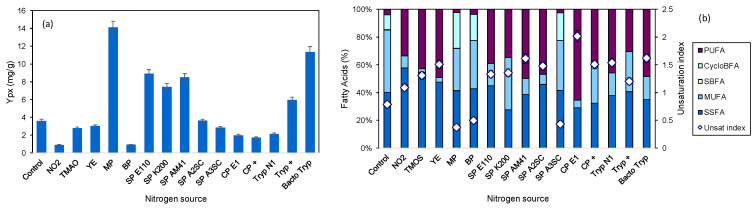
Influence of different nitrogen sources on Y_px_ (**a**) and fatty acid profile of the cellular membrane of *S. rubidaea* (**b**). Abbreviations: control (MB not supplemented); nitrite (NO_2_); yeast extract (YE); meat peptone (MP); bacteriological peptone (BP); soy peptone (SP); casein peptone (CP); tryptone (Tryp).

Several types of peptones, tryptones and nitrogen compounds, commonly found in the ocean, were added to the commercial MB. Figure 4 shows the influence of the nitrogen sources added on product yield (a) and on the lipid profile of the cell membrane (b).

The product-to-biomass yield decreased when both inorganic nitrogen sources tested, nitrite (NO_2_) and trimethylamine *N*-oxide (TMAO), were added to MB (Figure 4a). NO_2_ inhibited both cellular growth and product production in comparison to the control, resulting in a decrease to 25.7% in Y_px_. TMAO, which is a colorless amine oxide that accumulates in the tissue of marine animals in high concentrations and protects them against the adverse effects of temperature, salinity, high urea and hydrostatic pressure [46], decreased to 77.1% the Y_px_ in comparison to MB alone (Figure 4a). Three of the soy peptones allowed good results in Y_px_, doubling the value observed with MB. The highest Y_px_ values were attained with meat peptone (MP) and Bacto^TM^ tryptone (Becton, Dickinson and Company, Franklin Lakes, NJ, USA) with a more than 3-fold increase in comparison to MB alone. Since MP allowed the highest Y_px_ at 14.1 mg/g, it was selected as a nitrogen supplement in the remaining experiments.

When the cells grew on MB supplemented with most of the nitrogen sources tested, they produced PUFAs in significant amounts (Figure 3b). Exceptions were observed when the cells grew on MP-, BP- and SP A3SC-supplemented media, with the cells showing similar lipid profiles to the control cells with increased production of MUFAs and CycloBFAs.

The selected sources of both C and N were tested simultaneously to evaluate *S. rubidaea* growth conditions and product production. An equal concentration of 5 g/L of both sources was found to be ideal, leading to a 1.8-fold increase in biomass and product concentrations.

#### 2.1.3. Effect of Metal Ions

Taking into consideration the geochemical characteristics and chemistry of the thermal water emerging at the sea level in the place where this *S. rubidaea* strain was isolated from [47], the effect of selected metal ions, namely aluminum, arsenic, cobalt, chromium, copper, iron and nickel, added to MB was assessed on the production yields of this strain. The inhibitory effect of several first transition metal ions on both cell growth and pigment synthesis in *S. marcescens* was previously reported [48]. In the present study, the metal ions were added at a concentration of 0.1 g/L to the already present 0.1 g/L of Fe(III) in MB. The concentration selected is not considered toxic for marine organisms for any of the salts tested [49,50,51,52].

**Figure 5:**
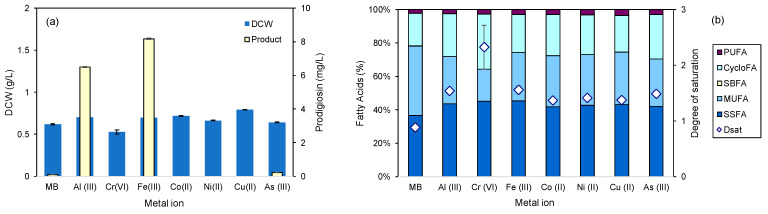
Influence of 0.1 g/L of different metal ions in supplemented MB on (**a**) the product per biomass yield after 24 h and (**b**) the lipid profile of the cells.

The cell dry weight of *S. rubidaea* increased ca. 13% when Fe(III) and Al(III) were added to MB, in comparison to when no supplementation was made, and 28% when Cu(II) was added (Figure 5a). Cells increased the production of prodigiosin 75.5-fold when MB medium was supplemented with Fe (III), whereas Al(III) and As(III) induced 59.9- and 2.1-fold increases, respectively (Figure 5a). Al(III), As(III) and Fe(III) are the most abundant ions, of those tested, in the water of the sampling site from where this *S. rubidaea* was isolated, Ferraria thermal springs in S. Miguel island, the Azores, Portugal [28,47]. Their natural release into the aquatic environment from the Earth’s crust occurs as a result of the hydrothermal vent linked to the Sete Cidades Volcano [47,50]. Fe(III) was selected for supplementing MB in the remaining studies due to its capacity to induce higher biomass and prodigiosin yields in *S. rubidaea*. Nevertheless, Al(III) could also be a good supplement since its toxicity decreases when bound to organic matter and at alkaline pH (e.g., 8.5) [50], being also a known co-adjuvant in vaccines [53]. The remaining metal ions tested did not cause an inhibitory effect on cell growth, with the exception of Cr(VI) which resulted in a 15% decrease in CDW (Figure 5a). In accordance with this, similar responses were observed when comparing the lipid profile of the cells grown in MB supplemented with different metal ions, with the exception of the Cr(VI)-supplemented medium: an average degree of saturation of 1.4 was observed, which is a 1.6-fold increase in comparison with cells grown without supplemented metal ions (Figure 5b). When grown in Cr-supplemented MB, the cells presented a degree of saturation of 2.3: the energy expended for the changes observed could be an explanation for the lower CDW observed (Figure 5).

To assess which inorganic iron source would be more available for *S. rubidaea*, iron(III) sulfate, iron(III) chloride and iron(II) chloride were tested with MB also supplemented with NaG and MP. Fe(III) is the most common form of iron in the ocean, but compounds with iron in this ionization state are sparingly soluble and/or in a form that is hardly metabolized. In the present study, iron(III) sulfate and iron(III) chloride were used since they are the most abundant forms in which iron is present in the oceanic hydrothermal plumes and vents, being scattered mainly in the form of volcanic ash [47,54,55,56]. Both forms are also used as ocean fertilizers [57]. Fe(II) was also used in the present study to assess which oxidation state of iron is best for inducing prodigiosin production. The Y_px_ values of the three types of inorganic iron salts used, Fe_2_(SO_4_)_3_, FeCl_3_ and FeCl_2_, were 94.9, 83.6 and 62.6 mg_prodigiosin_/g_DCW_, respectively. Iron sulfate was thus chosen as the metal ion for complementing MB, and the results indicate the preference for Fe(III) by the *S. rubidaea* cells.

When increasing concentrations of iron(III) sulfate, from 0.1 to 1 g/L, were added to MB, which already contained 0.1 g/L of iron(III) citrate, it was found that 0.2 g/L Fe_2_(SO_4_)_3_ allowed an 8-fold increase in Y_px_. Concentrations higher than this led to a decreased yield probably due to an inhibitory effect. This concentration was thus chosen for the remaining assays.

#### 2.1.4. Succinic Acid and Glycine

Looking further into the biosynthetic route of tetrapyrrole, compounds such as porphyrins, succinic acid and glycine appear as precursors of 5-aminolevulinic acid (5-ALA), a tetrapyrrole biosynthesis intermediate [58]. According to Sasaki et al. [59], a medium supplemented with 10–50 mM of succinic acid and glycine increased the production of 5-ALA.

**Figure 6:**
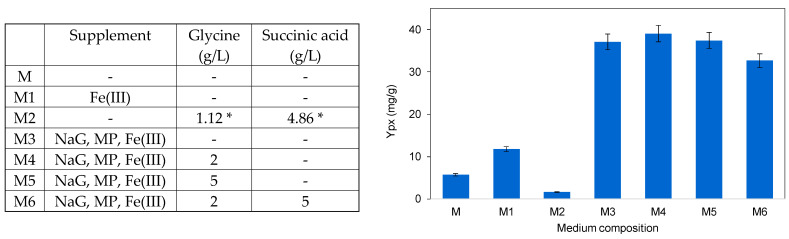
Effect of glycine and succinic acid, added as supplements to the carbon and nitrogen sources in MB, on the product-to-biomass yield of *S. rubidaea*. * Values adapted from [58].

To assess the effect of succinic acid and glycine on *S. rubidaea* cells, they were added to MB and MB supplemented with NaG, MP and Fe(III) at different concentrations (Figure 6). When 1.12 g/L glycine and 4.86 g/L succinic acid were added to MB, a 3.5-fold decrease was observed in comparison with MB, and a 7.3-fold decrease occurred when compared to the cultivation in MB supplemented with 0.2 g/L Fe_2_(SO_4_)_3_ (Figure 6). As previously mentioned, when MB was supplemented with NaG, MP and Fe(III), *S. rubidaea* cells significantly improved prodigiosin production (M3), and a 5% increase could be attained by adding 2 g/L glycine (M4). However, adding 5 g/L glycine (M5) showed no improvement in prodigiosin production, and adding 5 g/L succinic acid to 2 g/L glycine-supplemented MB (M6) resulted in a 12% decrease in production. In 1963, Shrimpton et al. used [2-^14^C]glycine incorporation to study prodigiosin production in *S. marcescens* and demonstrated that the methyl carbon atom of glycine is incorporated into both halves of the prodigiosin molecule with equal efficiency [60]. Additionally, it was shown that the 5-ALA, a specific precursor of porphyrins, is not used by *S. marcescens* in the formation of prodigiosin. Our results also indicate that 5-ALA should not participate in the biosynthetic pathway of prodigiosin in *S. rubidaea* since glycine only slightly improved the production of prodigiosin and the addition of succinic acid decreased its production. Considering the bioprocess economics, and these results, the following experiments were performed without the presence of succinic acid or glycine.

In the experiments described in the following sections, MB supplemented with 5 g/L NaG, 5 g/L MP and 0.2 g/L Fe_2_(SO_4_)_3_, and referred to as MB+S, was used.

#### 2.1.5. Effect of Salinity

**Figure 7:**
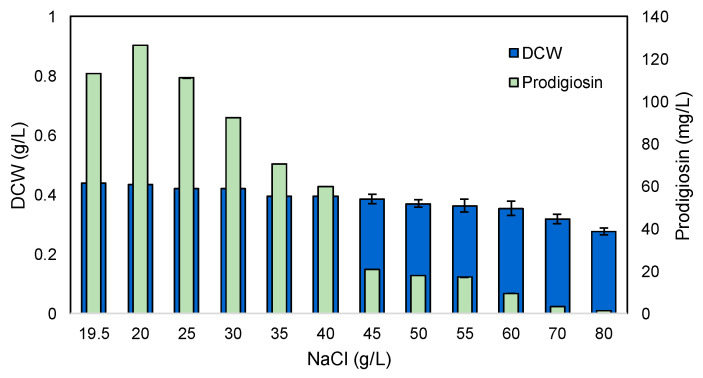
Effect of different concentrations of salt on cell growth and prodigiosin production.

The *S. rubidaea* strain used in this study was isolated, as mentioned, at Ferraria, where thermal water emerges at sea level [28]. The water is salty, resulting from the mixing of acidic brackish water at 100 °C and seawater from the Atlantic Ocean, and the electrical conductivity is equivalent to about 50% of the average seawater mineralization [47]. Additionally, seasonal fluctuations occur, as well as fluctuations during the day due to low and high tides. To assess the optimal concentration of NaCl for cell growth and prodigiosin production, a 24-well MTP was used with MB+S. To the initial 19.4 g/L of NaCl present in commercial MB [61], concentrations of NaCl ranging between 20 g/L and 70 g/L were tested (Figure 7).

A dose-dependent decrease in the concentration of biomass, measured after 24 h of cultivation, was observed with increasing concentrations of NaCl (Figure 7). Under osmotic stress caused by high salt concentrations, bacterial cells such as *Rhodococcus erythropolis* adapt their membrane fatty acid composition by increasing the percentage of PUFAs to decrease the entrance of NaCl through ionic channels and balance the osmotic pressure by accumulation, either by uptake from the environment or by de novo synthesis, of osmotic protectants like amino acids [20,34]. Similar adaptations were observed with *S. rubidaea,* with a ca. 37% reduction in DCW being observed between 19.5 and 80 g/L NaCl, indicating an ability of the cells to cope with more than double the average salt concentration of seawater. However, the prodigiosin concentration was maximum at 20 g/L of NaCl, and a steep decrease was observed with increasing salt concentrations, reaching only 18% at 45 g/L (Figure 7). As showed by Gallardo et al. [20], prodigiosin is a salt-dependent pigment, but unlike the *Vibrio* sp. isolated from a Chilean saline lake which can produce prodiginines at 100 g/L of NaCl, this *S. rubidaea* strain decreased prodigiosin production significantly at 70 g/L. Overall, the concentration of NaCl in commercial supplemented MB already allows the optimal production of both DCW and prodigiosin.

#### 2.1.6. Effect of Oxygen Availability

Oxygen availability is one of the most critical parameters for microbial growth and metabolite production. Insufficient oxygen supply is one of the problems associated with aerobic microbial cultures [62]. Microorganisms use the dissolved oxygen in the cultivation medium, but its concentration may be too low for microbial biosynthesis of specific metabolic products. In addition to oxygen limitation, oxidation/reduction mechanisms existing in the fermentation mixture may exert a chemical influence on the chemical structure of the products. In shaken flasks, oxygen limitation is common due to the low water solubility of oxygen at 20–40 °C and because oxygen supply is interrupted during the sampling procedures to monitor cell growth; the culture may not be able to recover from the interrupted aeration [63,64].

To determine the optimal oxygen concentration for increasing product productivity, three different liquid/headspace ratios were tested in shake flasks. The maximum growth rate was observed for 80% headspace, and a reduction to 91 and 86% was attained for 60 and 40% headspace, respectively. The maximum concentration of prodigiosin was also attained at 80% headspace.

A simple way of promoting a good distribution of air in the bulk media in a shake flask without changing medium volume is by varying the stirring speed and the shaker orbit diameter [65], the latter being out of the scope of this article. Using a fluorometric probe, the oxygen mass transfer coefficient (*k_L_a*) was determined in 500 mL shake flasks with and without baffles.

**Figure 8:**
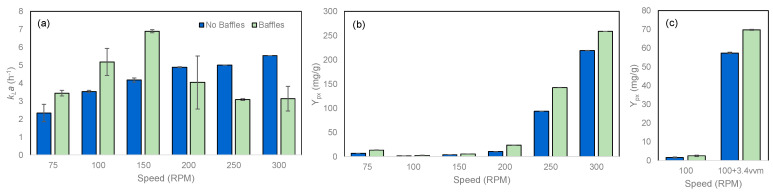
Effect of stirring speed on oxygen mass transfer coefficient, *k_L_a*, determined without cells (**a**), Y_px_ (**b**) and Y_px_ with forced aeration (**c**). These tests were carried out at 30 °C, in MB+S. Unbaffled (■) and bottom-baffled (■) 500 mL Erlenmeyer flasks.

In unbaffled shake flasks, the *k_L_a* values increased with shaking speed (Figure 8a). When baffled flasks were used, a bell-shaped curve was observed with a maximum *k_L_a* value at 150 rpm (Figure 8a). The value reached at 300 rpm was similar to those attained at 250 and 75 rpm. By geometrically changing the shake flask, with the introduction of baffles at the bottom, the central vortex formed by the orbital agitation was disrupted above 200 rpm, limiting oxygen transfer from the headspace to the liquid.

Y_px_ increased with increasing stirring speed in both unbaffled and baffled flasks, in particular at agitation speeds higher than 200 rpm (Figure 8b). Although the *k_L_a* was kept constant at 250 and 300 rpm in baffled flasks, product production per cell increased at the same level as that observed in unbaffled flasks. However, the relation between prodigiosin production and oxygen availability could be further demonstrated by adding forced air into the flasks: at 100 rpm, a 39- and 29-fold increase was observed in unbaffled and baffled flasks, respectively, when 3.4 vvm of air was added (Figure 8c).

Medium viscosity, which varies accordingly with composition and temperature, also affects oxygen availability transferred to the cells. A growth medium usually behaves as a Newtonian fluid, since it contains trace elements or complex components such as yeast extract at low concentrations, and the oxygen transfer in this medium is directly related to agitation intensity [66]. However, an increase in medium viscosity will limit the dissolved oxygen available, as shown by cultures with mycelium fungi and high cell density cultures [67]

**Figure 9:**
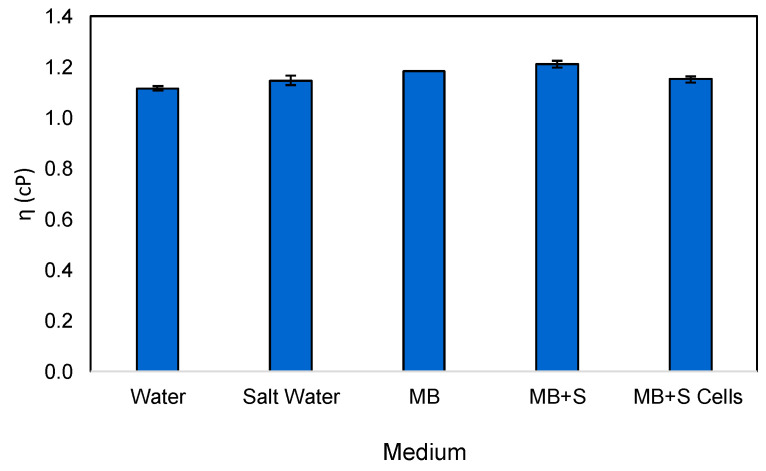
Viscosity of different media at 30 °C. Influence of cell presence on the medium viscosity is also shown.

To determine the viscosity of the medium developed and how its composition may affect growth in terms of the dissolved oxygen demands, Figure 9 shows the viscosity of several media at 30 °C at an increasing shear rate value, from 0 to 800 s^−1^.

The viscosity of the different mediums tested did not change with the increasing shear rate. However, the addition of different components to water increases its viscosity. MB is a good substitute for marine salt water due to their highly similar viscosity values. Interestingly, in a shake flask, where the cell concentration is usually not as high as that in a bioreactor, it is possible to observe that cell presence changes the medium viscosity, making it less viscous. This is not only due to component consumption but also to the changes in the cellular envelope and the formation of by-products such as prodigiosin.

#### 2.1.7. Growth Temperature

**Figure 10:**
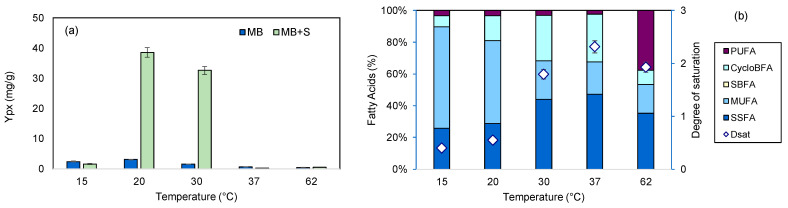
Influence of temperature on *S. rubidaea* growth in MB and MB+S. (**a**) Y_px_ obtained at 24 h of cultivation; (**b**) fatty acid profile of the cells grown in MB+S.

To evaluate how media engineering could be affected by temperature, the growth of *S. rubidaea* cells in supplemented MB was carried out at 15–62 °C and compared with the results published previously [28]. Supplemented MB allowed a significant increase in Y_px_ at 20 and 30 °C (Figure 10a). Unlike in MB, where the cells have an increased production of prodigiosin at 20 and 15 °C, when grown on supplemented MB, the production of prodigiosin was highest at 20 and 30 °C (Figure 10a). At 37 and 62 °C, the yields were similar in both media compositions tested. The modified medium also influenced the FA profile of the cells (Figure 10b). As the temperature rose, the amount of SSFAs and CycloBFAs increased, with a maximum being observed at 37 °C, and a concomitant decrease in MUFA and PUFA content was observed. The degree of saturation was half the value observed with only MB [28]. At 62 °C, a substantial increase in PUFAs was observed. Although these fatty acids have lower melting points, they could contribute to increase membrane order and stabilization, which should help the cells to grow at high temperatures [35].

#### 2.1.8. Effect of Light Exposure

Solar radiation can influence the growth of some heterotrophic bacteria. *S. marcescens* may use prodigiosin for storage of visible light energy, although light induces the phototranslation of prodigiosin [68]. However, prodigiosin has already been described as being able to provide protection against UVA and UVB light [69]. The beneficial effects of light in processes such as photosynthesis, photo-repair and photosensing can also severely harm living organisms through the formation of reactive oxygen species and/or other free radicals. Evolution has led to the development of a large set of ingenious light-induced signal-transduction response pathways that allow organisms to detect unfavorable light conditions and react appropriately [70,71].

To assess the effect of light on *S. rubidaea*, we grew the cells under conditions similar to those found near the shallow-water hydrothermal vent from where it was isolated. This was particularly important since the sampling site is exposed to solar radiation, particularly during low tide. MB and 100 mL^−1^ L shake flasks were used for cell cultivation to simulate different light paths since light intensity decreases with distance from the source and when traveling through liquids.

**Figure 11:**
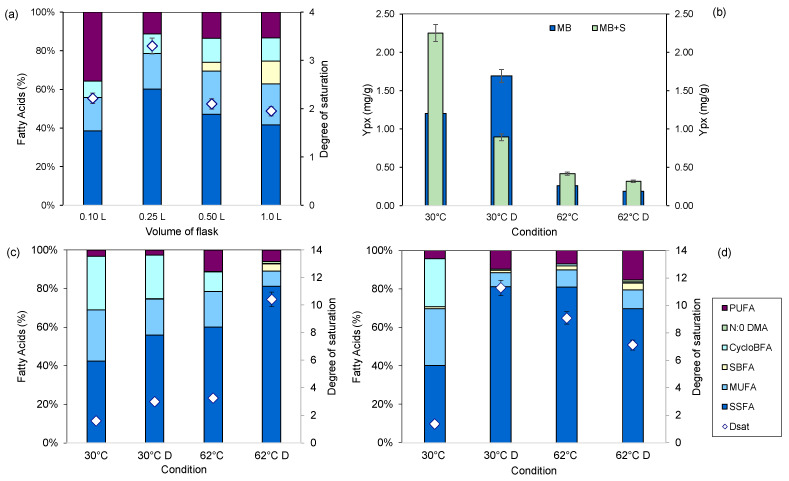
Effect of light, temperature and size of cultivation flask on the lipid profile and prodigiosin yield of *S. rubidaea* cells. Fatty acid profile of cells grown for 24 h in Erlenmeyer flasks with different volumes at 62 °C (**a**) and at 30 and 62 °C under light and dark conditions on MB (**c**) and supplemented MB (**d**). Prodigiosin-to-biomass yield under light and dark conditions at 30 and 62 °C (**b**). Under dark (D) conditions, the shaken flasks were covered with aluminum foil.

In small exposed areas, as in 0.1 L flasks, the cells produced equivalent amounts of SSFAs and PUFAs. As the exposed area and light distance increased, the cells showed lower contents of PUFAs as an increased amount of SSFAs was produced (Figure 11). In 0.5 L and 1 L Erlenmeyer flasks, the cells also produced SBFAs. Interestingly, the degree of saturation of the cell membranes in the larger flask was similar to that of the cells grown at 0.1 L. The cells thus changed their lipid composition in response to light exposure in the shaken flasks.

When daylight/night cycles were tested at 30 and 62 °C in *S. rubidaea* cells growing in MB and MB+S, it was found that the cells presented higher product-to-biomass yields in MB at 30 °C in the absence of light (Figure 11b). However, in supplemented MB medium, better product-to-biomass yields were observed under light conditions, both at 30 and 62 °C. Additionally, the cells increased the degree of saturation of the fatty acids under dark conditions, in comparison to the corresponding daylight condition (Figure 11c). In supplemented MB, the cells stopped producing CycloBFAs when grown in the dark and at 62 °C (Figure 11d). Curiously, cells grown at 30 °C in the dark in supplemented MB presented a lipid profile similar to cells grown at 62 °C. The additional changes observed under dark conditions and at 62 °C could be a reason why Y_px_ decreased in comparison to cells grown under light and at 30 °C.

### 2.2. Product Inhibition

The purpose of prodigiosin and its function in the survival of the *Serratia* strain remain to be fully understood [72]. To assess the effect induced upon the *S. rubidaea* cells, regarding biomass and product production, prodigiosin was initially added to growth media. Purified prodigiosin, already produced by this strain, was dissolved in absolute ethanol and added to the medium at concentrations of 57.3 µM, 28.6 µM and 5.7 µM. Absolute ethanol without prodigiosin was used for “0 µM” in a proportion of 10% of the total volume used.

**Figure 12:**
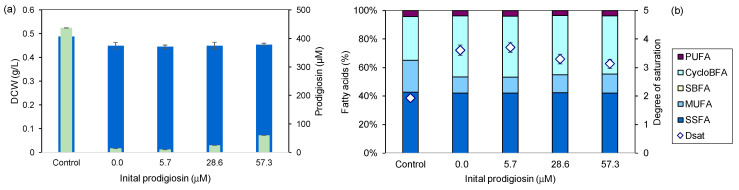
Inhibitory effect of prodigiosin on *S. rubidaea* cells. (**a**) DCW (■) and prodigiosin concentrations (■) and (**b**) lipid profile of cells in medium containing prodigiosin. Bacterial growth was carried out at 30 °C, 200 rpm, for 24 h in MB+S. The control was made only with MB+S.

The presence of absolute ethanol and prodigiosin mainly affected prodigiosin production. The cells were able to grow to similar DCW values in all tested concentrations of prodigiosin and in the presence of ethanol, showing only an 8% reduction, on average, in comparison to control cultures. Prodigiosin production, however, was reduced, on average, by 99% (Figure 12a).

The FA profiles of the cells show that the cells significantly increased the degree of saturation as a result of a decrease in MUFAs, in comparison with control cells (Figure 12b). Additionally, the content of CycloBFAs increased ca. 38%. A dose-dependent decrease in the degree of saturation from 3.6 to 3.1 was observed with increasing prodigiosin concentration. We hypothesize that the cells can recognize, probably by quorum sensing [73], the presence of prodigiosin, which is not recognized as a harmful molecule. Since it was already present in the medium, *S. rubidaea* cells did not produce prodigiosin.

### 2.3. Application of Supplemented MB in Serratia marcescens Cultures

As a proof of concept, the medium developed was tested with another marine *Serratia.* An *S. marcescens* strain we isolated from another location in Portugal was grown both in MB and MB+S and compared with *S. rubidaea.* The *S. marcescens* strain was isolated from a sample collected in a rock pool which could be regarded as an extreme environment due to the high exposure to sunlight, which led to seawater evaporation and a total salt concentration of ca. 60 g/L.

**Figure 13:**
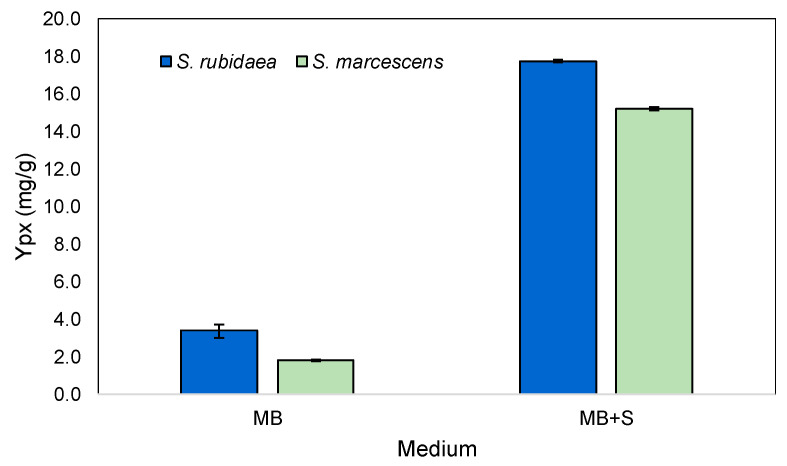
Y_px_ of different marine *Serratia* species in MB and MB+S.

The medium developed for *S. rubidaea*, when used with *S.* marcescens, led to the production of high yields of prodigiosin (Figure 13). In this case, *S. rubidaea* and *S. marcescens* increased prodigiosin production 8- and 13-fold, respectively. By changing the composition of the medium through optimization, increased production of prodigiosin in shake flasks could be achievable.

### 2.4. Scale-Up to 2 L Bioreactor

**Figure 14:**
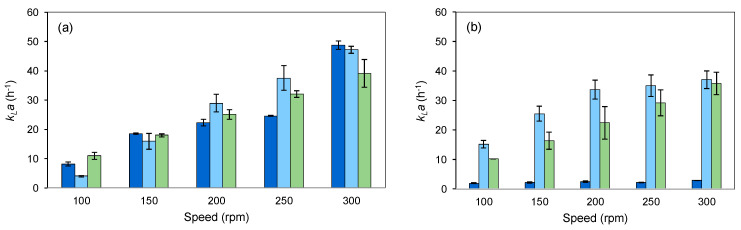
Variation in *k_L_a* with stirring speed in shake flask (■) and Electrolab (BE) (■) and Infors (BI) (■) bioreactors, using (**a**) double-distilled water and (**b**) 30 g/L NaCl solution.

To scale up the bioprocess to a 2 L bioreactor, maintenance of *k_L_a* was the scale-up criterion chosen. To keep this parameter along scales and geometries, the gassing-out static method was used to determine the *k_L_a* values obtained both in shake flasks and in bioreactors. Figure 14 shows the *k_L_a* values obtained in a range between 100 and 300 rpm, at 30 °C, using double-distilled water and a 3% NaCl solution.

**Figure 15:**
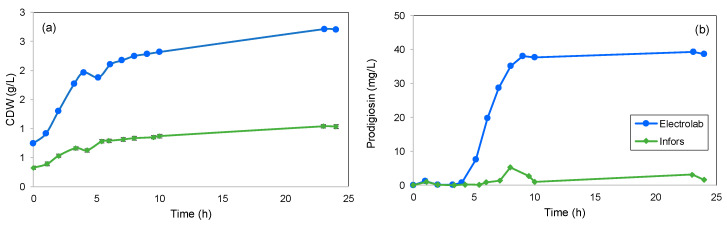
*S. rubidaea* growth (a) and prodigiosin production (b) obtained with supplemented MB. Agitation was changed from an initial 300 rpm to 200 rpm at the 6th h of fermentation. Temperature, pH and aeration were kept at 30 °C, 7.2 and 1 vvm, respectively.

The presence of salt affected the oxygen dissolved concentration in all bioreactors tested since it is known that salt decreases the solubility of oxygen in water. A comparison of both bioreactors revealed that the BE allowed a better aeration of the medium in the agitation range tested (Figure 14b). The difference in the geometry of the two bioreactors may account for this difference: the BE has a flat bottom and a height/diameter ratio of 1.9, whereas BI has a round bottom and a height/diameter ratio of 2.4. By comparing the results in Figure 14, it is possible to observe that the *k_L_a* values obtained in the bioreactors are 10 times higher than those in shake flasks. However, there is a similar behavior between the shake flask and the BE within the range of 200 to 300 rpm, the agitation speed at which prodigiosin starts to be produced in shake flasks. By applying the conditions used in the shake flasks to the two types of 2 L bioreactors used, the bioprocess was scaled up. Figure 15 shows the DCW and product production obtained in a two-stage 24 h fermentation in both bioreactors, and in Table 1, the corresponding growth parameters and productivities are presented.

Since no control was imposed upon the dissolved oxygen in the bioreactors, changes in agitation speeds were performed by manual input. Initially, the fermentations started with a stirring speed of 300 rpm to promote, like in the shake flasks, a rapid growth of biomass. This condition also allowed the production of prodigiosin to start at the 5th h, an hour earlier than what was already described for this strain [74,75]. At the 6th h, the speed was changed to 200 rpm so that the sheer stress felt by the cells, which were already in the deceleration stage of the exponential phase, would be lower.

Unlike prodigiosin production (Figure 15b), biomass concentration in both bioreactors was very similar during cultivation time (Figure 15a). The difference between fermentations may be due to the difference in vessel geometry. As shown in [76], the geometry characteristics of the BI, with its round bottom, offer a better mixing of the medium but lead to lower *k_L_a* values (Figure 14b). At 300 rpm, the conditions present were not sufficient to promote product formation. With this two-stage approach applied to the bioreactors, the biomass and product production achieved in the BE, offered, in relation to the shake flask, a 8-fold increase in Y_px_, corresponding to a prodigiosin production of 293.1 mg/L.

**Figure 16:**
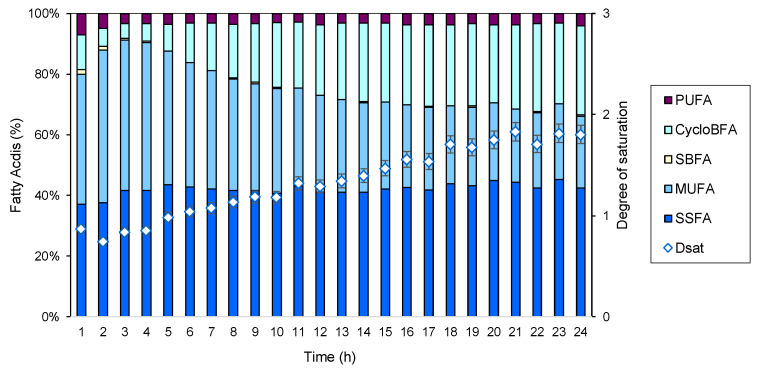
Fatty acid profile of *S. rubidaea* cells during 24 h fermentation in the BE.

A 24 h fermentation was carried out to assess the condition of the cells in the BE under the imposed operating conditions. With samples being taken every hour, an analysis of the lipid profile of the membrane of the cells was performed (Figure 16). This allowed for a better understanding of the cellular adaptation to the fermentation conditions. The *S. rubidaea* cells maintained the percentage of SSFAs throughout the fermentation time. During the exponential phase, the cells increased their MUFA percentage up to the 4th h of fermentation. This gives more flexibility to the cellular membrane, probably favoring a quick cellular division. At the 5th h, the product started to be produced intracellularly, and this coincided with an observed decrease in the content of MUFAs in the cellular membrane which was simultaneously accompanied by an increase in CycloBFAs. Although these CycloBFAs increase membrane fluidity, they confer higher stability to the membrane than MUFAs since they are more ordered [35]. CycloBFAs are usually produced in larger quantities during the stationary phase, as observed in this study. As the cells reach the stationary phase, and growth ceases, and de novo fatty acid synthesis is no longer possible; the FA profile of the cells is kept, as also observed. Curiously, the growth stage of the cells can be easily followed by the degree of saturation of their membranes (Figure 16).

## 3. Materials and Methods

### 3.1. Medium Composition Optimization

#### 3.1.1. Conical Tubes (15 mL)

First, 15 mL sterile conical tubes were filled with 60% of their volume with either MM supplemented with 30 g/L of NaCl (MM) [77] or MB. Each set of tubes was supplemented with 5 g/L of different carbon sources: sugars such as D-(+)-glucose monohydrate, sucrose (both from Fisher Scientific, Loughborough, UK), arabinose, fructose (both from Merck, Darmstadt, Germany), D-(+)-maltose monohydrate, mannose, D-(+)-melezitose monohydrate, D-(+)-raffinose pentahydrate, D-(+)-trehalose dihydrate, xylose (all from Sigma-Aldrich, Sigma-Aldrich, St. Louis, MO, USA) and mannitol (from Panreac Applichem, Barcelona, Spain); the amino acid glutamic acid (Sigma-Aldrich, St. Louis, MO, USA) and its sodium salt, sodium glutamate monohydrate (Merck, Darmstadt, Germany); the polysaccharides starch (Merck, Darmstadt, Germany) and inulin (Sigma-Aldrich, St. Louis, MO, USA); the polysorbate surfactant Tween 80 (Merck, Darmstadt, Germany); and glycerol (Panreac Applichem, Barcelona, Spain). Inoculation of each tube was performed with an isolated colony of *Serratia rubidaea*, taken from marine agar plates incubated for 24 h. The tubes were incubated horizontally at 30 °C and 150 rpm for 24 h. The assays were performed at least in duplicate.

#### 3.1.2. Shaken Flasks

To improve biomass production (measured as dry cell weight, DCW) and prodigiosin yields, the addition of minerals and nutrients to MB was assessed. For this purpose, 100 mL Erlenmeyer flasks filled to 40% working volume were used with MB supplemented, independently, with 0.25% complex carbon sources (i.e., lipids), 0.5% different nitrogen sources, 0.01% different metal salts and different NaCl concentrations. The complex carbon sources used were the following: used alimentary oil, (Vita D’Or^®^, Rio de Mouro, Portugal), fish oil (Pharma Nord, Vejle, Denmark), virgin olive oil (Riquitos—lagar de azeite Lda., Viseu, Portugal) and soy oil (Azeol, Torres Vedras, Portugal). The nitrogen sources used were divided between organic sources and inorganic salts. The organic nitrogen sources used were the following: casein peptones, soy peptones and tryptones (all from Organo Technie, La Courneuve, France), bacteriological peptone (Oxoid, Hampshire, UK), meat peptone (Sigma-Aldrich), bacto tryptone (BD™ Difco™, Franklin Lakes, NJ, USA) and yeast extract (Liofilchem, Waltham, MA, USA). The nitrogen inorganic salts tested were NaNO_2_ (Fisher Scientific) and TMAO·2H_2_O (Sigma-Aldrich). The different metal ions tested were CoCl_2_·6H_2_O, CuSO_4_·5H_2_O, NiSO_4_·6H_2_O (all from Sigma-Aldrich), NaAsO_2_, Al_2_(SO4)_3_·18H_2_O, CrO_3_ (all from Merck) and Fe_2_(SO4)_3_ (Riedel-de Haën, Seelze, Germany). For the salt tolerance study, only NaCl (Panreac) was added to MB. The determination of the culture oxygen needs was performed in 500 mL volume Erlenmeyer flasks filled with 20%, 40% and 60% volume MB. In these experiments, the flasks were incubated at 30 °C with a stirring speed of 200 rpm in an Agitorb 200 orbital shaker (Aralab, Rio de Mouro, Portugal).

The environmental conditions tested, namely the influence of light and growth temperature, were also assessed in Erlenmeyer flasks filled with 40% of their total volume. For the temperature studies, 100 mL Erlenmeyer flasks were incubated at 15 °C and 20 °C in an Optic Ivymen System refrigerated orbital shaker (JP Selcta, Barcelona, Spain), while studies at 30, 37 and 62 °C were carried out in an Agitorb 200 orbital shaker (Aralab). Assays with Erlenmeyer flasks of different volumes, namely 250 mL, 500 mL and 1000 mL, were also performed at 62 °C. To assess the effect of light on cell growth and production, 250 mL Erlenmeyer flasks were incubated at 30 °C and 62 °C under light (provided by a Philips LED tube, 2300 lm), and a set was covered with aluminum foil to simulate dark conditions. In all experiments, each flask was inoculated with 10% of its working volume, using a cell suspension grown overnight, and stirred at 200 rpm. All assays were carried out at least in duplicate.

#### 3.1.3. Oxygen Monitoring

Oxygen concentration was monitored in real time using microtiter plates (MTPs) with sensor spots. To determine the optimum concentration of C and N sources, as well as NaCl concentration, for supplementing the MB, standard 24-well plates (Sarstedt, Nümbrecht, Germany) with 1.5 mL volume per well were initially used. For the comparison between different marine *Serratia* sp. fermentations in the developed medium (marine broth plus supplements, MB+S), the dissolved oxygen concentration was monitored online in 24-well OxoDish^®^ plates (from PreSens Precision Sensing GmbH, Regensburg, Germany) with 1.5 mL volume per well. According to the manufacturer, the Oxodish^®^ microtiter plates have a resolution of ±0.4% O_2_, a precision of ±1% O_2_ at 20.9% O_2_, and a drift <0.2% O_2_ within one week. The dissolved oxygen concentration data were acquired in real time by the SDR_v37 software (also from PreSens).

The inoculation of each well was carried out with an overnight-grown culture, and the volume of the cell suspension added was the volume necessary to achieve an initial optical density (OD) at 600 nm of 0.1 in each well (the final volume per well was kept at 1.5 mL). The plates were incubated at 30 °C and 200 rpm in the previously mentioned Agitorb 200 orbital shaker. All assays were performed at least in duplicate, with dry cell weight (DCW) and product production being measured at the end of the experiment.

### 3.2. Medium Viscosity

To study the dynamic viscosity of the different media used, a parallel plate Rheometer MCR 92 (Anton-Paar GmbH, Graz, Austria), operating in rotational and oscillatory modes, was used, and data were analyzed with the RheoCompass™ software (also from Anton-Paar; version 1.32.258), and 1 mL samples were taken in order to fill the 1 mm gap of the CP50 geometry. All experiments were carried out at 30 °C, in duplicate, with an increasing shear rate ($\dot{\gamma }$) until 800 s^−1^.

### 3.3. Bioreactors

Bacterial growth was performed in four 2L bioreactors, with different geometries: two Fermac 360 bioreactors (Electrolab, Gloucesteshire, UK) with a tank diameter close to the tank height and a plain bottom; two Minifors bioreactors (Infors HT, Basel, Switzerland) with a tank height that is double the tank diameter and a round bottom. The Fermac bioreactors contained 1.5 L of medium, while the Minifors bioreactors were filled with 1.2 L. Air was provided by a compressor, and the bioreactors contained oxygen, pH and temperature probes, allowing the control and monitoring of these parameters.

### 3.4. Oxygen Transfer

#### 3.4.1. *k_L_a* Determination in Shake Flasks

To determine the *k_L_a* in unbaffled and baffled 500 mL Erlenmeyer flasks, the dynamic gassing-out method was used [78,79]. These flasks were filled with 40% liquid of (i) water, (ii) a 30 g/L NaCl solution or (iii) the supplemented MB (MB+S). The flasks were agitated at different stirring speeds ranging from 75 to 300 rpm. The deaeration step was performed by bubbling nitrogen in the medium, while reaeration occurred as a result of surface aeration due to agitation of the flasks which were closed with cellulose stoppers. The oxygen inside the liquid was monitored online using a PSt3 fluorometric probe (from PreSens Precision Sensing GmbH). According to the manufacturer, the PSt3 probe has a response time of 6 s, a resolution of ±0.4% O_2_ and a precision of ±0.1% O_2_ at 20.9% O_2_, and a drift <0.03% O_2_ within one month.

#### 3.4.2. *k_L_a* Determination in 2 L Bioreactors

The determination of the *k_L_a* in the bioreactors was performed using the same method as in the shake flasks. Polarographic probes (Mettler Toledo, Greifensee, Switzerland) were used to measure the dissolved oxygen in the bioreactors. The four bioreactors with 2 L capacity, two Fermac 360 (BE) bioreactors and two Minifors (BI) bioreactors, were filled with 1.5 and 1.2 L, respectively, of water and with a 30 g/L NaCl solution. The deaeration and reaeration steps were performed with nitrogen and compressed air, respectively. The air flow into the bioreactors and temperature were maintained constant at 1 vvm and 30 °C. The stirring speed was changed between 100 and 300 rpm.

### 3.5. Biomass and Product Production in 2 L Bioreactors

The conditions allowing the highest biomass and product production in shaken flasks and the determined *k_L_a* values were used to grow batch cultures, using MB+S, in the 2 L bioreactors. Bacterial cells were grown for 24 h at 30 °C, using 1 vvm of aeration, and pH 7.2 which was adjusted with the addition of 1.5 M H_2_SO_4_ and 2 M NaOH. The bioreactors were stirred at 300 rpm during the first 6 h and at 200 rpm during the remaining time.

### 3.6. Phenotypic Adaption of Cells at the Lipid Level

To assess the influence of both the different composition alterations made to the MB and the environmental conditions tested upon the cells, the fatty acid composition of the cellular membranes was determined. During the 24 h growth, 1 mL of culture medium was harvested every hour. The cell lipid composition was determined as previously mentioned [80]. Briefly, the cell samples were centrifuged at 12,500× *g* for 5 min (HERMLE Labortechnik GmbH, Wehingen, Germany) and washed with Milli-Q water. After another centrifugation at 12,500× *g* for 5 min and removal of the supernatant, the fatty acids (FAs) from the cellular membranes were extracted and simultaneously methylated to fatty acids methyl esters (FAMEs) using the Instant FAME^TM^ method from MIDI (MIDI, Inc., Newark, DE, USA) [81]. The FAMEs were determined by gas chromatography analysis on an Agilent Technologies 6890 N gas chromatograph (GC, Agilent, Santa Clara, CA, USA), with a flame detector and a 7683 B series injector, using a 25 m Agilent J&W Ultra 2 capillary column. The FAMEs were identified by the Sherlock^®^ software version 6.2 using the PFLAD1 method (from MIDI) and also by using calibration standards. At least two independent cultures, grown under each tested condition, were used, and the presented results are the average of the lipid extractions and analyses. The degree of saturation of the fatty acids of the cellular membrane was defined as the ratio between total saturated and total monounsaturated fatty acids, whereas the unsaturation index was defined as the sum of the percentage of each unsaturated fatty acid multiplied by the number of double bonds in the molecule.

### 3.7. Product Extraction

During cell growth, product production was monitored as previously described [28]. Briefly, 1 mL samples of cell suspension were taken from the culture medium and centrifuged at 12,500× *g* for 8 min. The resulting pellets were washed with 1 mL of Milli-Q water, followed by a new centrifugation cycle, and the supernatant was discarded. The washed pellets were resuspended in a 3:1 (*v*/*v*) solution of ethyl acetate/acetic acid glacial (both from Sigma, St. Louis, MO, USA) and left protected from light for 1 h at room temperature. After centrifugation at 7000× *g* for 5 min, the supernatants were analyzed by UV-VIS spectroscopy between 200 and 800 nm.

### 3.8. Analytical Methods

#### UV-VIS Spectroscopy

Both cellular growth and prodigiosin production were monitored offline as previously described [28]. In summary, the optical density (O.D.) was measured at 600 nm, and the spectrum between 200 and 800 nm was recorded to assess product production using a Multiskan Go UV-VIS spectrophotometer microplate and cuvette reader (Thermo Scientific, Waltham, MA, USA). Dry cell weight (DCW) was determined from the O.D. measurements using a calibration curve (periodically confirmed by placing samples at 65 °C for 24 h and weighting the cells), while product concentration was determined by a calibration curve relating it with absorption at 535 nm.

## 4. Conclusions

With the application of medium engineering, the yields of biomass and prodigiosin produced by the marine *S. rubidaea* increased 2-fold and 8-fold, respectively, in shake flasks. MB was supplemented with different carbon and nitrogen sources, as well as metal ions, different salt concentrations and prodigiosin precursors, and the effect upon the cells was studied using high-throughput systems. It was found that this marine *S. rubidaea* strain grew faster and to higher cell densities when sodium glutamate, meat peptone and Fe(III) were added to MB. The increase in biomass and product resulted from glutamate being a precursor of prodigiosin [82], meat peptone’s similarity with the bacterial peptone present in MB, and iron being an essential component that probably helped the biochemical route of prodigiosin production.

One of the most important conclusions of this study is that the tested *S. rubidaea* strain was able to use numerous carbon and nitrogen sources while adapting the lipid composition of the cellular membrane to several induced changes. This could be the result of adaptation to the extreme environment from which it was isolated. The shallow hydrothermal vent is located in the Atlantic Ocean, and it is largely affected by tidal conditions, which result in, e.g., temperature, pH, salinity and nutrient variation between high and low tides. The ability to adapt to continuously changing conditions thus helped the strain performance during the different assays.

The effects of environmental conditions, such as temperature, light conditions, and oxygen availability, on prodigiosin production were also assessed. The supplementation of MB resulted in the cells changing their prodigiosin production optimum temperature from 15–20 °C to 20–30 °C. Normal day/night cycles led to higher production of prodigiosin than dark conditions, and high oxygen concentrations were necessary to maintain cellular growth and the oxidative state of the product. By supplementing MB, its applicability as a suitable medium for growing different marine prodigiosin-producing Serratia strains, with high yields of DCW and prodigiosin, was established.

The bioprocess could be scaled up, from shaken flasks to 2 L bioreactors, using *k_L_a* maintenance as the scale-up criterion. A two-stage fermentation, where agitation was changed from 300 rpm to 200 rpm after 6 h, was performed in two 2 L bioreactors with different geometries using the supplemented medium. The geometry of the vessel greatly influenced the behavior of the cells by affecting dissolved oxygen and was, apparently, the main factor responsible for the differences observed in growth and production in the two bioreactors. In a flat-bottom bioreactor, it was possible to achieve a volumetric productivity for biomass and product of 113.05 mg DCW/(L.h) and 12.21 mg prodigiosin/(L.h), respectively, with *S. rubidaea*. In terms of Ypx, there was an 8-fold increase relative to shake flasks.

The environmental tenacity of the studied *S. rubidaea* strain, with its window for growth and production spanning from at least 15 to 62 °C and from 19 to 80 g/L NaCl, in the presence of metal ions such as Al(III), Fe(III) and As(III) suggests that it may be a robust strain for industrial application. The numerous carbon and nitrogen sources that the bacterium may metabolize also indicate that strains from extreme environments could have an adaptative advantage over those from regular environments for biotechnological applications.

## Figures and Tables

**Table 1:** *S. rubidaea* growth parameters and productivities in both bioreactors used. BE—Electrolab bioreactor; BI—Infors bioreactor.

Bioreactor	µ_max_ (h^−1^)	T_d_ (h)	P_r_^Biomass^ (mg/(L.h))	P_r_^Prodigiosins^ (mg/(L.h))
BE	0.28	2.52	113.05	12.21
BI	0.22	3.16	43.25	1.63

## Data Availability

The data presented in this study are available on request from the corresponding author.
